# Trait-based responses to cessation of nutrient enrichment in a tundra plant community

**DOI:** 10.1007/s00442-021-05064-w

**Published:** 2021-10-29

**Authors:** Chhaya M. Werner, Maria Tuomi, Anu Eskelinen

**Affiliations:** 1 Department of Physiological Diversity, Helmholtz Center for Environmental Research (UFZ), 04318 Leipzig, Germany; 2 German Centre for Integrative Biodiversity Research (iDiv) Halle-Jena-Leipzig, 04103 Leipzig, Germany; 3 Department of Ecology and Genetics, University of Oulu, 90014 Oulu, Finland; 4 Department of Arctic and Marine Biology, UiT, The Arctic University of Norway, 9019 Tromso, Norway

**Keywords:** Nutrient addition, Recovery, Tundra, Functional traits, Litter

## Abstract

Plant communities worldwide show varied responses to nutrient enrichment—including shifts in species identity, decreased diversity, and changes in functional trait composition—but the factors determining community recovery after the cessation of nutrient addition remain uncertain. We manipulated nutrient levels in a tundra community for 6 years of nutrient addition followed by 8 years of recovery. We examined how community recovery was mediated by traits related to plant resource-use strategy and plant ability to modify their environment. Overall, we observed persistent effects of fertilization on plant communities. We found that plants with fast-growing traits, including higher specific leaf area, taller stature and lower foliar C:N, were more likely to show a persistent increase in fertilized plots than control plots, maintaining significantly higher cover in fertilized plots 8 years after cessation of fertilization. Additionally, although graminoids responded most strongly to the initial fertilization treatment, forb species were more vulnerable to fertilization effects in the long-term, showing persistent decline and no recovery in 8 years. Finally, these persistent fertilization effects were accompanied by modified environmental conditions, including persistent increases in litter depth and soil phosphorous and lower soil C:N. Our results demonstrate the potential for lasting effects of nutrient enrichment in nutrient-limited systems and identify species traits related to rapid growth and nutrient-use efficiency as the main predictors of the persistence of nutrient enrichment effects. These findings highlight the usefulness of trait-based approach for understanding the persistent feedbacks of nutrient enrichment, plant dynamics, and niche construction via litter and nutrient build-up.

**Supplementary Information:**

The online version contains supplementary material available at 10.1007/s00442-021-05064-w.

## Introduction

Nutrient enrichment has been implicated as a driver of biodiversity loss and compositional change for plant communities globally (Bobbink et al. 2010; Harpole et al. 2016; Payne et al. 2017). As the negative impacts of nutrient enrichment on biodiversity have become clear, suggestions for ways to decrease nutrient inputs on natural systems have gained traction, including the abandonment of agriculture (Bakker and Berendse 1999), international management plans to reduce nutrient loads (Nielsen et al. 2019), or active restoration through top-soil removal and liming (Smolders et al. 2008). Despite these efforts, patterns of recovery after the cessation of nutrient enrichment have thus far been less well studied. In some cases, soil nutrients can recover relatively quickly to pre-enrichment levels (Stevens 2016), while the effects on plant communities in experimental studies often last even decades after treatments ceased, sometimes but not always including depressed species richness (Strengbom et al. 2001; Isbell et al. 2013; Street et al. 2015; Stevens 2016). Further investigating which mechanisms lead to either recovery or persistent changes after cessation of nutrient enrichment are needed to improve our understanding of community processes following global change pressures.

Theoretical predictions and field data indicate that in nutrient-enriched conditions, traits that allow plants to take advantage of increased resources are those associated with higher competitive ability for light in resource-rich conditions by promoting fast growth (Reich 2014). These “resource-acquisitive” traits include high relative growth rate, high specific leaf area, high leaf N and P concentrations (Westoby et al. 2003; Adler et al. 2013; Reich 2014), and taller stature, which is associated with higher competitive ability for light (Fargione and Tilman 2002; Hautier et al. 2009). In contrast, species with the opposite, “resource conservative” traits that permit survival and growth under nutrient-constrained conditions are less likely to be able to take advantage of increased nutrient availability (Chapin et al. 1986; Fargione and Tilman 2002; Adler et al. 2013), and can exhibit greater loss-likelihood in resource-rich conditions (Stevens et al. 2011; Helsen et al. 2014; Kidd et al. 2017; Kaarlejärvi et al. 2017). Consequently, trait-driven species responses to nutrient addition can lead to shifts in functional trait composition, decreased species richness, and ultimately altered ecosystem functioning (Suding et al. 2008; Cadotte et al. 2011).

The potential role of traits in the recovery or persistent responses of species and communities after nutrient enrichment remains to be explored. On one hand, since species with resource-acquisitive traits are often most responsive to nutrient limitation (Chapin et al. 1986), they could be outcompeted by resource-conservative species once nutrient levels were no longer artificially elevated (Tilman 1988). However, if resource-acquisitive species modify their environment via niche construction, the abundance of these species may change the physical and chemical conditions of their environment in a way that promotes their dominance even after the cessation of nutrient enrichment (Laland et al. 2016). Niche construction can result in an environment where light is the most limiting resource, for which they are often dominant competitors (Hautier et al. 2009; Reich 2014; Borer et al. 2014). Resource-acquisitive species are likely to produce more litter due to higher turnover of leaves (Adler et al. 2013), which can reinforce community changes via a physical barrier to seed germination or light reduction (Lamb 2008). The litter produced by fast-growing species is also often rich in nitrogen and decomposes faster (Cornwell et al. 2008), which could maintain nutrient-rich conditions that benefit these same fast-growing, nutrient-demanding species. Despite the great potential of traits for predicting various community processes, their role as predictors of species’ persistence in previously nutrient-enriched conditions has yet to be addressed.

Here we present results from a long-term study on the patterns and drivers of persistent consequences of nutrient enrichment for plant communities. This study in tundra experimentally manipulated nutrient availability with 6 years of nutrient addition, crossed factorially with habitat type, and reported trait-dependent changes in diversity and productivity (Eskelinen et al. 2012). We then examined whether the effects of nutrient enrichment persisted 8 years after the cessation of the nutrient addition treatment by comparing the pretreatment communities to communities 14 years after the experiment was established (i.e. showed persistent responses) or whether the communities had returned to the pretreatment stage (i.e. had recovered). Furthermore, we assessed whether species’ persistence in previously nutrient-enriched conditions was explained by traits. Tundra ecosystems are ideal to test theories of persistent nutrient effects, with strong resource limitation, and high diversity of species in small plots (Shaver and Chapin 1980; Virtanen et al. 2013). These communities have few to no exotic species, which allows tests of the general effects of nutrient enrichment on native communities (separate from feedbacks between invasive species and nutrient enrichment). Finally, the interspersion of two different habitat types with greatly varying initial nutrient levels at our study site (Eskelinen et al. 2009) facilitates investigation of the generalizability of patterns and mechanisms.

We hypothezed that the previously fertilized plots would have persistently lower species richness (H1a), higher turnover of species from the pre-nutrient addition community (H1b) and larger shifts in functional groups (H1c) than control plots. We predicted that species with resource-acquisitive traits (i.e., greater SLA, height and N concentrations) would be more likely to have persistent increases in the enriched plots than those with resource conservative traits (H2). We also predicted that this trait-based increase would be associated with aspects of niche construction, including increased accumulation of litter (H3a) and persistent differences in soil nutrients (H3b).

## Methods

### Study system

This study was conducted in tundra communities above treeline on Mount Saana, located in northwest Finland (69.05′ N, 20.83′ E). Treeline lies in 600–650 m a.s.l. On Mt. Saana, two distinct bedrock types result in a mosaic pattern of soil and vegetation (hereafter referred to as “habitat types”). Non-acidic and relatively nutrient-rich soils, derived from dolomitic bedrock, support forb- and graminoid-rich *Dryas* heaths (“fertile habitat”). In contrast, acidic and relatively nutrient-poor soils lie above siliceous rocks, and support dwarf shrub- and graminoid-rich *Empetrum* heaths (“infertile habitat”). Fertile habitats had higher soil pH and NH_4_-N than infertile habitats (Eskelinen et al. 2009). For our experimental study, we chose five patches for each of these two habitat types, fertile and infertile habitats, spatially interspersed within a distance of 5 km, at altitudes of 720–800 m a.s.l. and with similar topographic and moisture conditions.

### Experimental design

In 2004, we established eight 25 × 25 cm plots at each of the ten sites (five fertile and five infertile habitats), for a total of 80 plots. To examine the persistent effects of nutrient enrichment on plant communities, we established a fully factorial combination of three treatments: fertilization, liming, and grazer exclusion. Each site had one replicate of each of the consequent treatment combinations. Our original experimental design included liming and grazer exclusion to make sure that lower pH at some sites would not constrain plants from responding to fertilization and because recovery from fertilization could depend on grazing (Olsen and Klanderud 2014).

Fertilization was conducted with a fast-dissolving NPK 16-9-22 fertilizer, which we applied twice per growing season (in mid-June and at the end of July) every year from 2005 to 2010. Fertilizer was applied in the plot and an additional 15 cm wide buffer on each side. Total nutrient quantities per year were 9.6 g N/m^2^, 5.4 g P/m^2^, and 13.2 g K/m^2^. Fertilizer applications were followed immediately by watering using 500 mL of water per plot from nearby brooks. Liming to manipulate pH was conducted on the same schedule and with the same spatial buffer as fertilization. We added 300 g/m^2^ of dolomite lime (CaMg(CO_3_)_2_) to the plots in 2005 and 600 g/m^2^ in each of 2006–2010. Grazer exclosures were established in August 2004 and constructed of galvanized mesh netting approximately 1.5 m in circular diameter (mesh size 1.2 × 1.2 cm). The exclosures were 80–100 cm above ground and 10 cm deep into the soil to prevent grazing by both semi-domesticated reindeer (the dominant grazer in the system), voles and lemmings. Fertilization and liming treatments were terminated after 2010, but grazer exclosures were left in place throughout the duration of the study.

We initially included liming treatment as a predictor variable in our models, but since it was not a significant component to any of the initial treatment effects (Eskelinen et al. 2012) or in any of our analyses, we pooled across the limed and un-limed plots and will not discuss this treatment further. Furthermore, reindeer grazing in the system was high from the start of the experiment until around 2012; however, due to changed reindeer herding practices and following shifts in the movement of the herds in Kilpisjärvi area, grazing pressure on Mt. Saana was considerably lower from 2013 to 2019. For this reason, grazing pressure after the termination of fertilization is not fully comparable to years 2004–2010. We included grazing in our statistical models as it did affect some variables; however, due to changed reindeer herding practices our results probably greatly underestimate the potential impact of grazing on recovery from fertilization and is therefore given less consideration.

### Community measurements

The presence and percent cover of all vascular species in the 25 × 25 cm plots was estimated during the peak biomass of vegetation in late July–August of 2004 (just before application of experimental treatments), 2010 (at the end of fertilization and liming treatments), and 2019. In 2004 and 2019 cover was estimated visually by an experienced person, and in 2010 cover estimates were conducted using a point-intercept method (Jonasson 1988) with 40 evenly spaced sampling points per 25 × 25 cm plot. Although we included the 2010 data as useful reference information for initial treatment effects, our questions and findings focus predominantly on the changes from 2004 to 2019 (persistent effects of treatments) when the same method was used. For four species pairs that were difficult to tell apart in their vegetative state, we pooled the cover estimates (*Carex bigelowii* and *C. vaginata*, *Equisetum arvense* and *E. pratense*, *Equisetum scirpoides* and *E. varigetum*, *Anthoxantum odoratum* and *Poa alpigena*).

In 2019 we additionally measured litter depth at three uniformly assigned points per plot, and sampled soil at three points in the 15 cm fertilized buffer zone surrounding each plot. Soils were later analyzed for total N and C (used to calculate C:N ratio), NH_4_, P, K, Ca, and Mg concentrations (Eurofins lab, Oulu).

### Trait measurements

To investigate the role of plant traits in species’ responses to fertilization, we measured three traits: specific leaf area (SLA, leaf area [mm^2^] per unit of dry leaf mass [mg]), foliar C:N ratio (based on the percentage of plant total carbon and nitrogen in plant leaves), and height (mm). Trait data were collected for 38 species in 2011 from the area where the experiment was carried out, with > 10 individuals collected for each species, following standard collection and handling protocols (Cornelissen et al. 2003). We additionally used trait data from a study located in a nearby area for six species (*Anthoxantum odoratum* and *Poa alpigena* [pooled as above], *Pyrola minor*, *Ranunculus acris*, *Gentiana nivalis*, and *Solidago virguarea*) which were collected in 2014 (Kaarlejärvi et al. 2017). SLA was analyzed using ImageJ to calculate fresh leaf area (Rasband 1997). Total C and N of leaves were analyzed on a CHN Element Analyzer (Fisons Instruments, Milan, Italy).

### Data analysis

Analyses were carried out using linear mixed-effects models (LME) using a model selection approach. The full models included habitat type, fertilization, herbivory, and their interactions as explanatory variables, and site as a random variable. We assessed differences in multiple vegetation metrics, considering both initial differences (comparing 2004 and 2010 data) and persistent differences (comparing 2004 and 2019 data). This structure was used to investigate initial and persistent differences on the plot level in species richness (H1a), species turnover (H1b, calculated as 1—Jaccard similarity), and changes in total graminoid, forb, fern, and shrub cover (H1c, only comparing 2004–2019). Since 2010 cover data was measured using a different method than 2004 and 2019 cover data, we did not compare it directly to the other years, but did model differences between treatments in 2010 functional group cover to demonstrate initial treatment effects (Supplement S3). We did not have pre-treatment data for moss and lichen cover, so we modeled this using the 2019 measurements only rather than a change between years (H1c). We also modeled litter depth (H3a) and soil nutrients (H3b) measured in 2019 as response variables. Model selection was conducted using comparisons of AICc values to select the best-fit model from all possible combinations, AICc values and ΔAICc comparisons are reported in Supplemental Table S1. Results are reported as effect sizes ± standard error.

To investigate plant traits as predictors of individual species’ responses to fertilization (H2), we took the difference in species’ percent cover between 2004 and 2019 and simplified this to a binary representation of change, either increasing or not increasing. We modeled this response using generalized linear mixed-effects models (GLMM) with a binomial structure, with a separate model for each trait (height, SLA, and C:N). The response variable was the probability for each species to increase in each plot, and the predictors were the traits (trait values were centered and scaled), habitat type, fertilization, herbivory, and their interactions (including three- and four-way interactions) as fixed factors and a site as a random variable. We used the same AICc model selection approach described in the previous paragraph. Using this GLMM structure allowed us to consider species that were lost from or colonized each plot relative to the local species pool. To ensure that we were focusing on species that were or could be present in plots, rather than those that are completely absent from a given site, only species that were present in the site at any of the three-time points were included in the analysis. As a robustness check, we also included a LME model of change in cover for the species present in the plot, the results of which were generally consistent and are included in Supplemental Table S2.

All data management and analyses were conducted in R and used ‘simba’, ‘plyr’, and ‘tidyverse’ packages (Wickham 2011, 2017; Jurasinski and Retzer 2012; R Core Team 2019). All models were fit using the ‘lme4’ package, and model selection was conducted using the ‘MuMIn’ package (Bates et al. 2015; Bartoń 2019). Plots of the fitted relationship between traits and probability of increase were generated using the glm.predict() function, which uses the delta method approximation for standard error estimates. Figures were made using ‘ggplot2’ and ‘cowplot’ packages (Wickham 2016; Wilke 2019).

## Results

### H1: community change

**Fig. 1:**
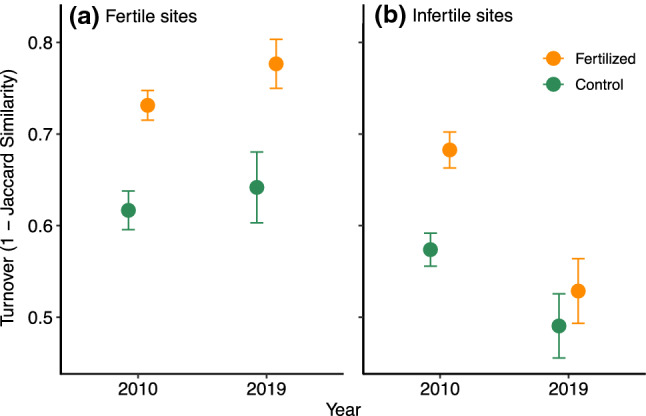
Species turnover compared to 2004 pre-treatment data, in **a** fertile and **b** infertile sites. Turnover is measured as 1—Jaccard similarity (using presence/absence data) between time points for each plot (mean ± SE). Color indicates fertilization treatment

Species richness initially increased more in fertilized plots than in unfertilized plots (i.e., comparing 2004–2010, Table 1; 1.3 ± 0.48), but showed no persistent effects of fertilization (i.e., comparing 2004–2019, Supplemental Fig. S1) despite an overall decrease in species richness across all treatments (from 9.9 ± 0.32 species per plot in 2004 to 8.1 ± 0.23 in 2019). We did find both initial and persistent effects of fertilization on species turnover (Fig. 1). Initial turnover between 2004 and 2010 was higher in fertilized plots than unfertilized plots across all treatments and habitats (0.15 ± 0.02). Herbivore presence mitigated the initial effects of fertilization on turnover but did not completely counteract it (Supplemental Fig. S2; − 0.078 ± 0.03). Persistent species turnover between 2004 and 2019 was also higher in fertilized plots than unfertilized plots (0.086 ± 0.03). There was no persistent interaction between fertilization and herbivory treatments.

**Table 1:** Effects of fertilizer, herbivory, habitat, and their interactions on richness change, community turnover (1—Jaccard similarity), change in graminoid cover, change in forb cover, 2019 moss and lichen cover, and 2019 litter depth

	Intercept	Fertilized	Herbivory	Habitat	Fertilized * herbivory	Fertilized * habitat	Herbivory * habitat
Richness change							
Initial	0.97 ± 0.41	1.3 ± 0.48	–	− 0.75 ± 0.48	–	–	–
Persistent	− 2.8 ± 0.54	–	0.72 ± 0.44	1.3 ± 0.70	–	–	–
Species turnover (Jaccard)							
Initial	0.59 ± 0.02	0.15 ± 0.02	0.045 ± 0.02	− 0.045 ± 0.02	− 0.078 ± 0.03	–	–
Persistent	0.66 ± 0.03	0.086 ± 0.03	–	− 0.19 ± 0.03	–	–	–
Graminoid change							
Persistent	− 11.8 ± 3.4	–	10.7 ± 3.7	11.1 ± 4.9	–	–	− 8.1 ± 5.2
Forb change							
Persistent	− 11.6 ± 6.4	− 40.8 ± 6.4	18.8 ± 6.4	15.9 ± 9.0	–	39.2 ± 9.0	− 17.4 ± 9.0
Moss cover							
2019	9.4 ± 1.6	− 5.0 ± 1.8	–	− 3.7 ± 1.8	–	–	–
Litter depth							
2019	2.2 ± 0.37	2.0 ± 0.46	− 1.8 ± 0.46	0.76 ± 0.33	− 1.6 ± 0.66	–	–

Values are effect size ± standard error, ‘–’ indicates that the variable was not included in the best model

**Fig. 2:**
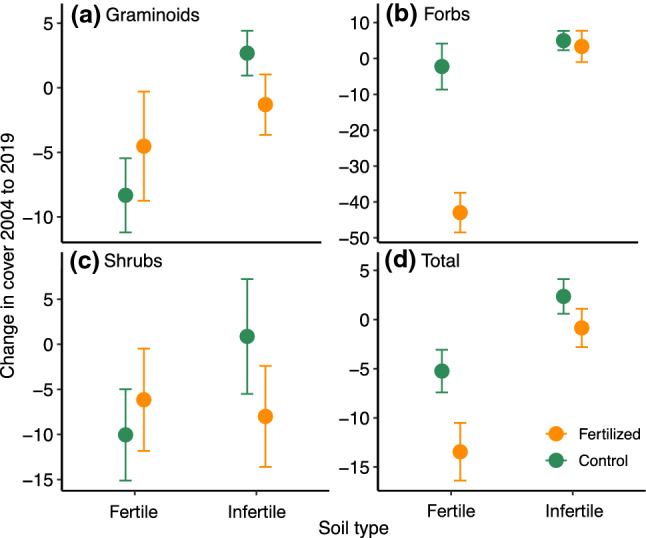
Change in cover from 2004 to 2019 of **a**–**c** different functional groups and **d** total cover, by soil type. Values are displayed as mean ± SE. Total cover also includes *Equisetum* species, which are not included in any of the three main functional groups

Although graminoid cover had a strong initial response to fertilization, with an average cover in 2010 of 74% in fertilized plots, compared to 27% in unfertilized plots (Supplement Fig. S3; 34 ± 5.5), there was no persistent difference of fertilization on graminoid cover, with 2019 graminoid cover averaging 14% in fertilized plots and 10% in unfertilized plots (Fig. 2a). (Note that these cover values are comparable within but not between years due to different methods of measuring cover in 2010 versus 2004 and 2019, see methods for details). In contrast, forb cover showed minimal initial response to fertilization, with an average cover in 2010 of 26% in fertilized plots and 23% in unfertilized plots (Supplement Fig. S3; 4.1 ± 2.6). However, we did observe persistent negative effects of fertilization on forb cover in fertile habitat (Fig. 2b, 19% versus 49%) but not infertile habitat (19% versus 18%; Table 1; habitat × fertilization 39 ± 9.0). There was an additional interaction between herbivory and habitat on persistent differences in forb cover (− 17 ± 9.0), but this did not interact with the fertilization treatment. Shrub cover did not change in response to fertilization (Fig. 2c, Supplemental Fig. S5). Species-level changes are presented in Supplemental Table S3. Moss and lichen cover (measured only in 2019) was lower in fertilized plots than unfertilized plots (2.5% versus 7.6%; Supplemental Fig. S4).

### H2: traits as predictors of species responses

**Fig. 3:**
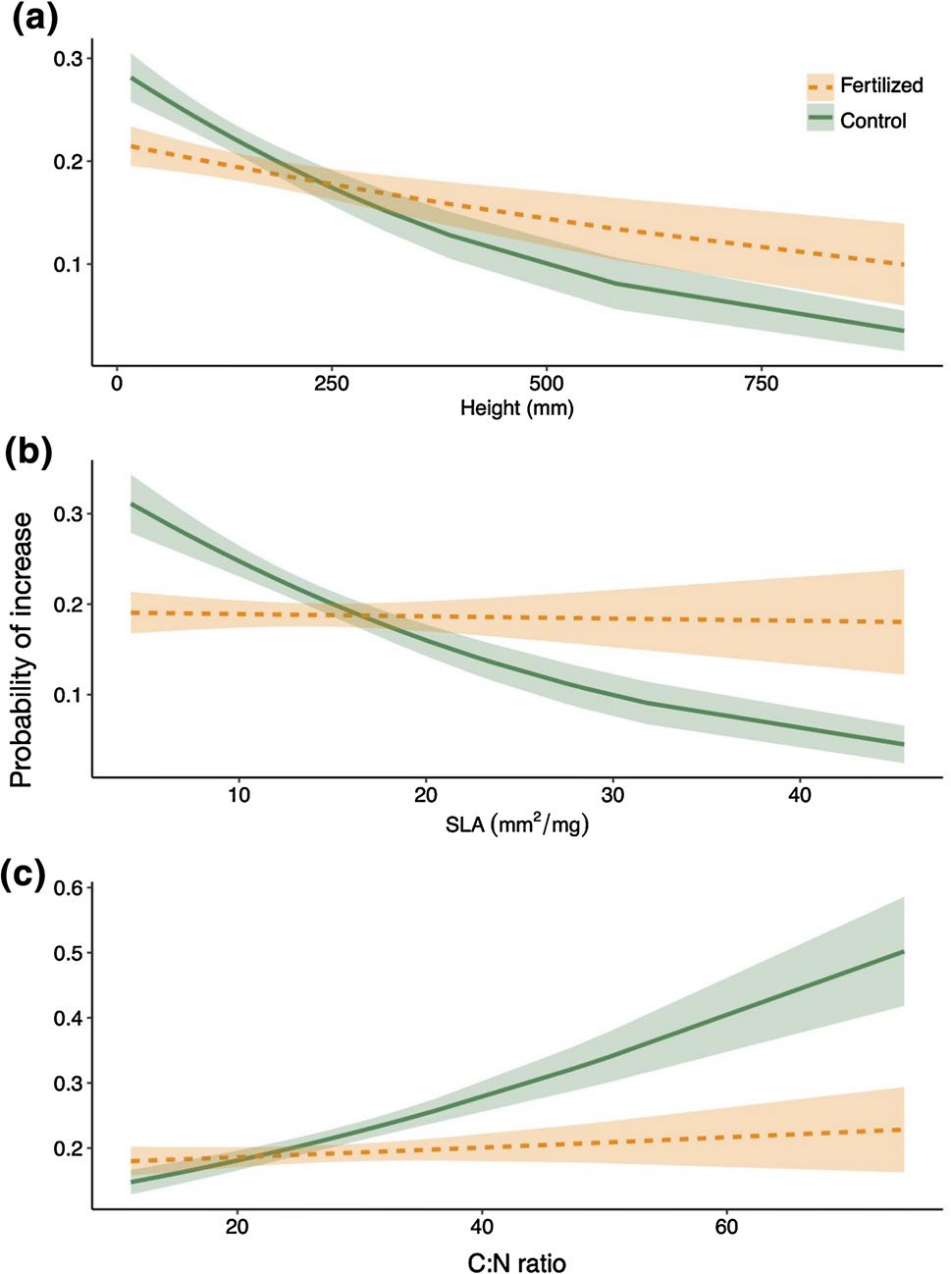
Relationship between species’ traits and their probability to increase in cover between 2004 and 2019 by fertilization treatment. Traits include **a** plant height **b** specific leaf area **c** leaf C:N. Values are displayed as fitted relationship with estimated standard errors, generated using the ‘glm.predict’ function for these models

We found that functional traits interacted with fertilization to explain the probability of species increasing in cover from 2004 to 2019, i.e., persistent responses (Table 2). Tall-statured species were less likely to increase overall, but more likely to increase in fertilized plots than control plots (Fig. 3, trait × fertilized effect size 0.27 ± 0.15). Species with low SLA were mostly likely to increase in control plots, while in fertilized plots species with high SLA were equally likely to increase as species with low SLA (trait × fertilized 0.39 ± 0.14). Species with a high C:N ratio—i.e., species having low N concentrations—were most likely to increase in control plots, while in fertilized plots species with low and high C:N ratio were equally likely to increase (Fig. 1c; trait × fertilized − 0.27 ± 0.12).

These relationships between traits and fertilization were consistent within habitats as well as across habitats (Supplemental Fig. S5), although the overall relationships between height or SLA and probability of increasing differed somewhat between habitat types, with a stronger advantage to tall-statured and high SLA species in-fertile sites (Supplemental Fig. S5; height trait × habitat − 0.29 ± 0.14; SLA trait × habitat − 0.26 ± 0.14). Although the grazer exclusion treatment had an overall effect on species’ probability to increase (all trait models 0.17 ± 0.11), this did not differ between fertilization treatments or interact with species traits.

### H3: litter depth and soil nutrients

**Fig. 4:**
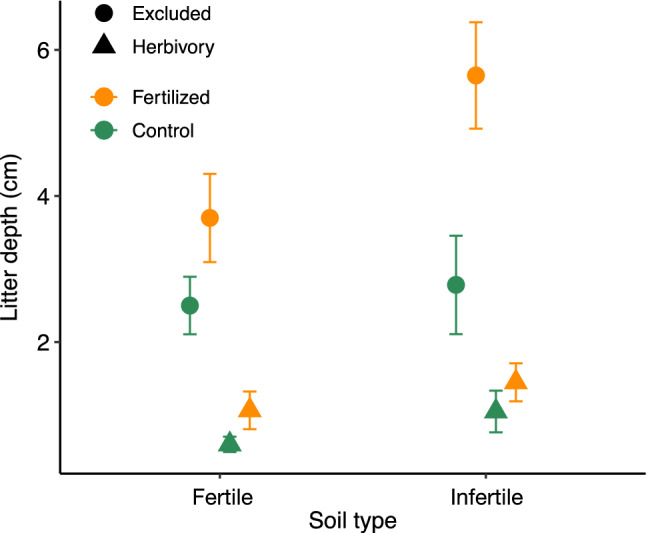
Litter depth by fertilization treatment and herbivory treatment, in fertile and infertile sites (mean ± SE). Litter depth was higher in fertilized and ungrazed plots

Litter depth was higher in fertilized plots (3.0 mm versus 1.7 mm; Fig. 4; 2.0 ± 0.46) and ungrazed plots (3.7 mm versus 1.0 mm; 1.8 ± 0.46) as well as infertile habitats (2.7 mm versus 2.0 mm; 0.76 ± 0.33). There was an additional interaction between fertilization and herbivory, with a larger effect of fertilization on litter depth in plots where herbivores were excluded than those where they were present (− 1.6 ± 0.66).

Soil phosphorus (P) concentration and soil C:N ratio indicated some persistent below-ground effects of fertilization, although no other nutrients differed by fertilization treatment (Supplemental Fig. S6). P was higher in fertilized plots than unfertilized plots (1200 ppm versus 1000 ppm; effect size 176 ± 44). C:N ratio differed strongly between habitat types (17 versus 22; 4.7 ± 1.2) but was marginally lower in fertilized plots across habitat types (19 versus 20; − 0.75 ± 0.37). Total nitrogen, ammonia, nitrate, potassium, calcium, and magnesium all showed no persistent effects of fertilization.

**Table 2:** Effect of fertilizer, herbivory, habitat, traits (height, specific leaf area, and foliar C:N) and their interactions on the probability of species increase between 2004 and 2019

	Intercept	Trait	Fertilized	Herbivory	Habitat	Trait * fertilized	Trait * habitat
Height	− 1.4 ± 0.11	− 0.30 ± 0.13	− 0.13 ± 0.11	0.17 ± 0.11	0.30 ± 0.11	0.27 ± 0.15	− 0.29 ± 0.14
SLA	− 1.5 ± 0.11	− 0.28 ± 0.12	− 0.07 ± 0.11	0.17 ± 0.11	0.20 ± 0.11	0.39 ± 0.14	− 0.26 ± 0.14
C:N	− 1.4 ± 0.11	0.31 ± 0.08	− 0.13 ± 0.11	0.17 ± 0.11	0.23 ± 0.11	− 0.27 ± 0.12	–

Values are effect size ± standard error, ‘–’ indicates that the variable was not included in the best model

## Discussion

Adding to the growing evidence that plant communities are slow to recover after nutrient addition (Strengbom et al. 2001; Isbell et al. 2013; Street et al. 2015; Stevens 2016), we found persistent differences in fertilized plant communities over 8 years after the end of the nutrient treatment. While several other studies have identified individual species or groups that showed lasting responses to fertilization (Olsen and Klanderud 2014; Street et al. 2015), ours is among the first to successfully use functional traits as predictors of these persistent changes. Furthermore, these changes were associated with persistent litter build-up and enhanced soil nutrients. These results support the predictions of niche construction theory (Thakur and Wright 2017), that species can promote their own abundance by modifying the environmental filters present in the local area (Laland et al. 2016), and provide evidence that this species-engineered microenvironment has strong consequences for the trait distribution of species in the community.

We found that species with traits associated with resource acquisitiveness (tall stature, high SLA, and low foliar C:N) were more likely to have persistent increases in fertilized plots than control plots. Similar suites of traits have previously been associated with species most likely to respond to nutrient addition (Suding et al. 2005; La Pierre and Smith 2015), but the role of traits in recovery and persistent changes has been less clear. Our findings indicate that species with resource-acquisitive traits may modify their environment to benefit their own success in ways that hamper community recovery to the pre-nutrient-enriched state. We found higher litter quantity in previously fertilized plots (up to twice as deep as the control plots) 9 years after the cessation of fertilization. Deep litter layers can inhibit germination (Kitajima and Tilman 1996; Henry et al. 2004; Lamb 2008), and reduce the immigration success of shorter-statured resource-conservative species, consequently maintaining the dominance of already established resource-acquisitive species. High SLA and low C:N, traits that in our data were associated with persistent responses to fertilization, are strongly linked to faster litter decomposition rates (Cornwell et al. 2008) which can combine with persistent microbial community changes to result in persistently elevated nutrient cycling rates (Carreiro et al. 2000; Power et al. 2006; Clark et al. 2009; Högberg et al. 2014; Gravuer and Eskelinen 2017; Bowman et al. 2018). Elevated nutrient cycling rates could in turn benefit resource-acquisitive plants that take advantage of resources as they become available (Suding et al. 2008), resulting in an overall fast-cycling system (Wardle et al. 2004; Eskelinen et al 2020). It is possible that such feedback mechanisms maintain the dominance of resource-acquisitive species in our system even 8 years after the cessation of nutrient enrichment. The reinforcement between above-ground traits and below-ground processes may represent alternative states created by nutrient enrichment; potentially transient states, but persistent on the scale of decades (Isbell et al. 2013; Chisholm et al. 2015).

In addition to trait-based changes, we observed persistent species turnover and changes in the total cover of different functional groups. During the initial period of nutrient enrichment, we observed elevated species richness and higher species turnover in fertilized plots. The effects on species richness did not persist after the cessation of nutrient addition, but the differences in species composition (higher turnover rates) did, indicating that the species recruiting into the fertilized plots after the end of the treatment were different than those that were lost. Deeper litter layer such as we observed in the previously fertilized plots could have particularly strong influence on forbs—the group that showed the largest persistent decline in our study. We observed a persistent drop in forb cover in-fertile habitats, with fertilized plots having an average of 19% forb cover compared to 49% in unfertilized plots. This difference is particularly striking because it was not apparent as a response to nutrient addition, but appeared as a lagged response to the nutrient enrichment 8 years after the treatments were terminated. In tundra communities, where strongly vegetatively reproducing shrubs and graminoids form a considerable proportion of species, seedling-based reproduction is often found for forbs (Welling and Fennici 2000; Austrheim and Eriksson 2003; Eskelinen et al. 2017). The deeper litter layer may have prevented the recruitment of forb seedlings after the cessation of nutrient addition. These litter layer impacts could also extend to bryophyte and lichen communities, suppressing their growth, as suggested by the approximately half lower total cover of bryophytes and lichens in control plots than in fertilized plots. We did find that graminoid cover returned to pre-treatment levels, which has been observed in some recovery experiments (Bowman et al. 2018) but not others (Olsen and Klanderud 2014; Street et al. 2015). Long-term plant community changes or direct effects of nutrients could also deplete belowground seed bank diversity, further reducing the potential for recovery (Bakker and Berendse 1999; Basto et al. 2015; Eskelinen et al. 2021, accepted).

The trait-based patterns we observed were generally consistent across the two habitat types we studied and across herbivory treatments. The interspersed tundra habitats differ not only in initial soil conditions but also in community composition, ranging from the dominance of very slow-growing evergreen dwarf shrubs in infertile heaths to N-rich forbs and legumes in fertile heaths (Eskelinen et al. 2009; Stark et al. 2012). Nevertheless, species’ responses in both habitats were explained by their traits in a similar way. Persistent effects of nutrient addition may also be influenced by mowing or grazing regime, as previous studies have found that removal of standing biomass (including live plants and litter) can improve recovery trajectories (Olff and Bakker 1991; Olsen and Klanderud 2014; Storkey et al. 2015). We did not observe effects of reindeer exclusion on any aspects of recovery other than litter depth; however, this is likely due to altered herd management and related changes in grazing intensity at our study site (see “Methods”), a caveat that cautions against drawing strong conclusions about this factor in our study. Further, we suspect that these changes in herd management and grazing pressure, coupled with climate trends that are particularly strong in arctic ecosystems (Box et al. 2019) but see Virtanen et al. (2021), are responsible for the strong changes we observed over 15 years in our control plots. It is noteworthy that even relatively low grazing pressure can mitigate the persistent effects of nutrient enrichment on litter accumulation, with possible consequences for community resilience through germination and above-belowground interactions. These habitat- and grazing regime-independent results highlight the strength of our findings and point toward the potential of plant traits to be a useful generalizable currency for understanding persistent responses to nutrient enrichment.

Plant traits have been used as a generalizable currency for understanding existing differences between communities (McGill et al. 2006), and for forecasting species and community vulnerability and resistance to various global change factors, including climate warming, changes in rainfall, and nutrient enrichment (Kimball et al. 2016; Bjorkman et al. 2018; Harrison and LaForgia 2019). We propose that they also have the potential in understanding community recovery from such changes, niche construction mechanisms and patterns of resilience and regime shifts between alternative states. Our results highlight the usefulness of trait-based approaches for predicting persistent responses to nutrient enrichment, with implications on planning restoration strategies in human-degraded ecosystems.

## Supplementary Information

Below is the link to the electronic supplementary material.

Supplementary file1 (PDF 532 kb)

## Data Availability

We have archived our data in a permanent Figshare repository at https://doi.org/10.6084/m9.figshare.14959863.v1.

## Code availability

We have archived our code in a permanent Figshare repository at https://doi.org/10.6084/m9.figshare.14959863.v1.
