# Supplementary material for: Trait-based responses to cessation of nutrient enrichment in a tundra plant community
Source: Oecologia. 2021 Oct 29;197(3):675–84. doi: 10.1007/s00442-021-05064-w (PMC8585805; doi:10.1007/s00442-021-05064-w)
Supplement: Supplementary file 1 — Supplementary file1 (PDF 532 kb) [file 442_2021_5064_MOESM1_ESM.pdf]

Supplemental Figures

Figure S1: Species richness through time, by habitat type and fertilization treatment. Values are displayed as mean ± S.E.

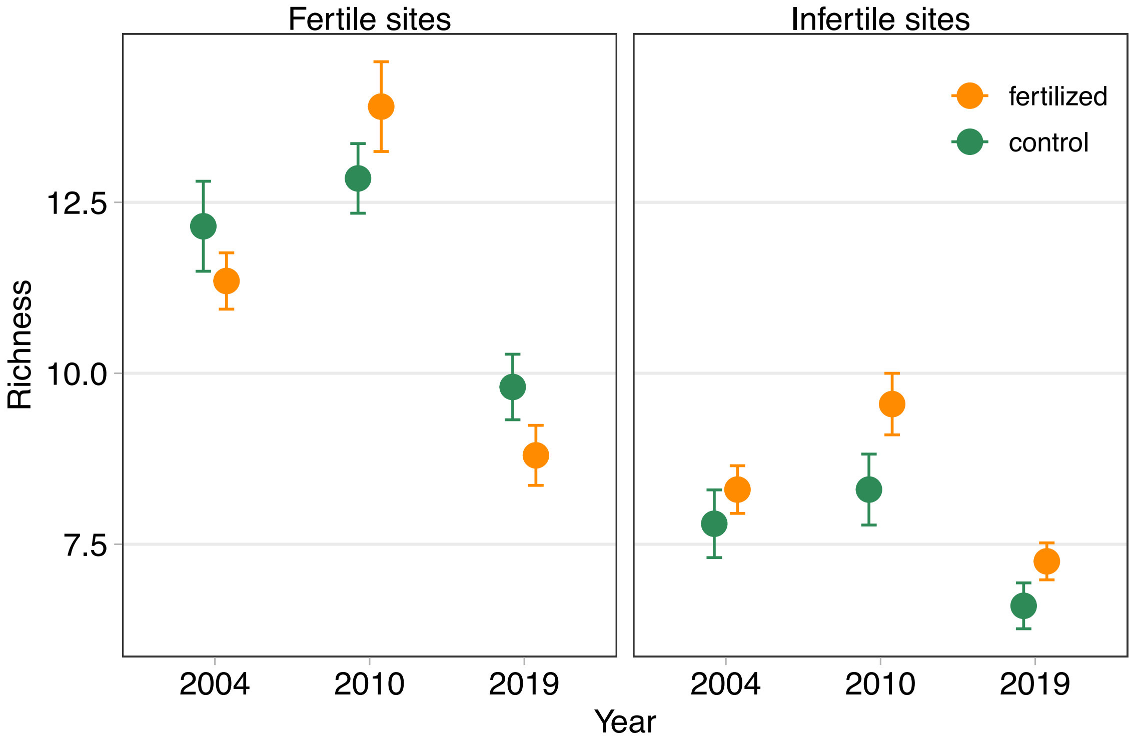

Figure S2: Effects of herbivory treatment on species turnover. Values are displayed as means ± S.E.

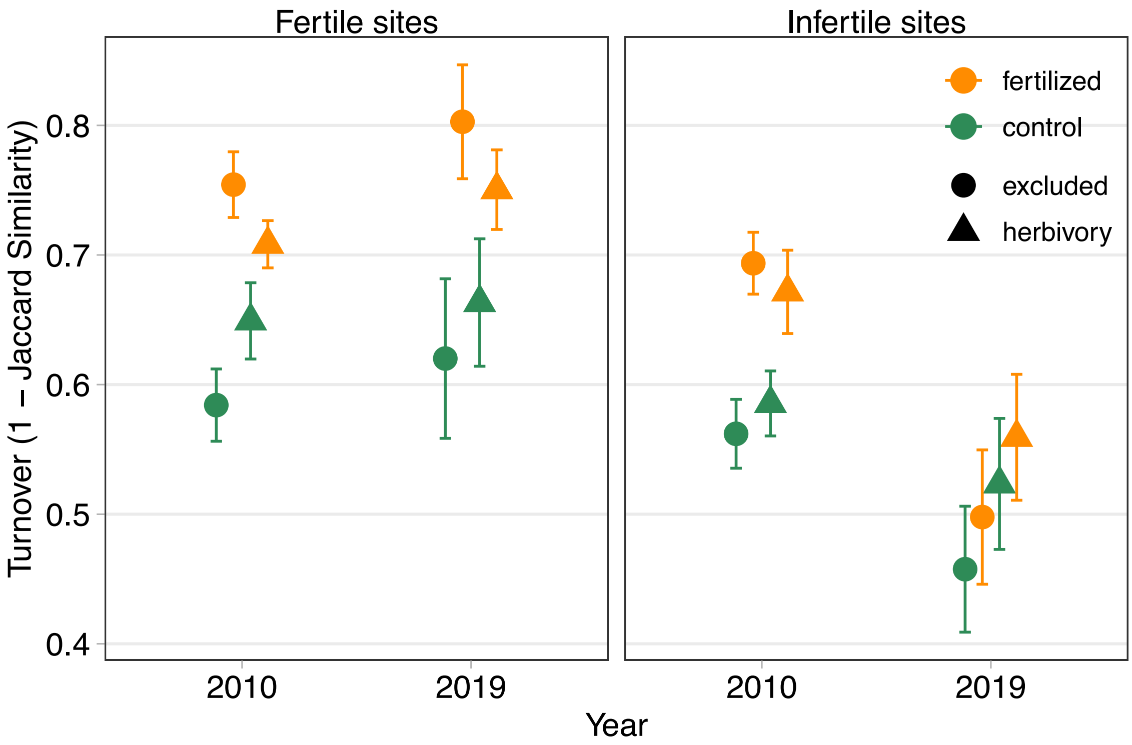

Figure S3: Cover of functional groups in 2010, measured using pin hits (2004 and 2019 cover were measured using visual estimates). Values are displayed as means ± se.

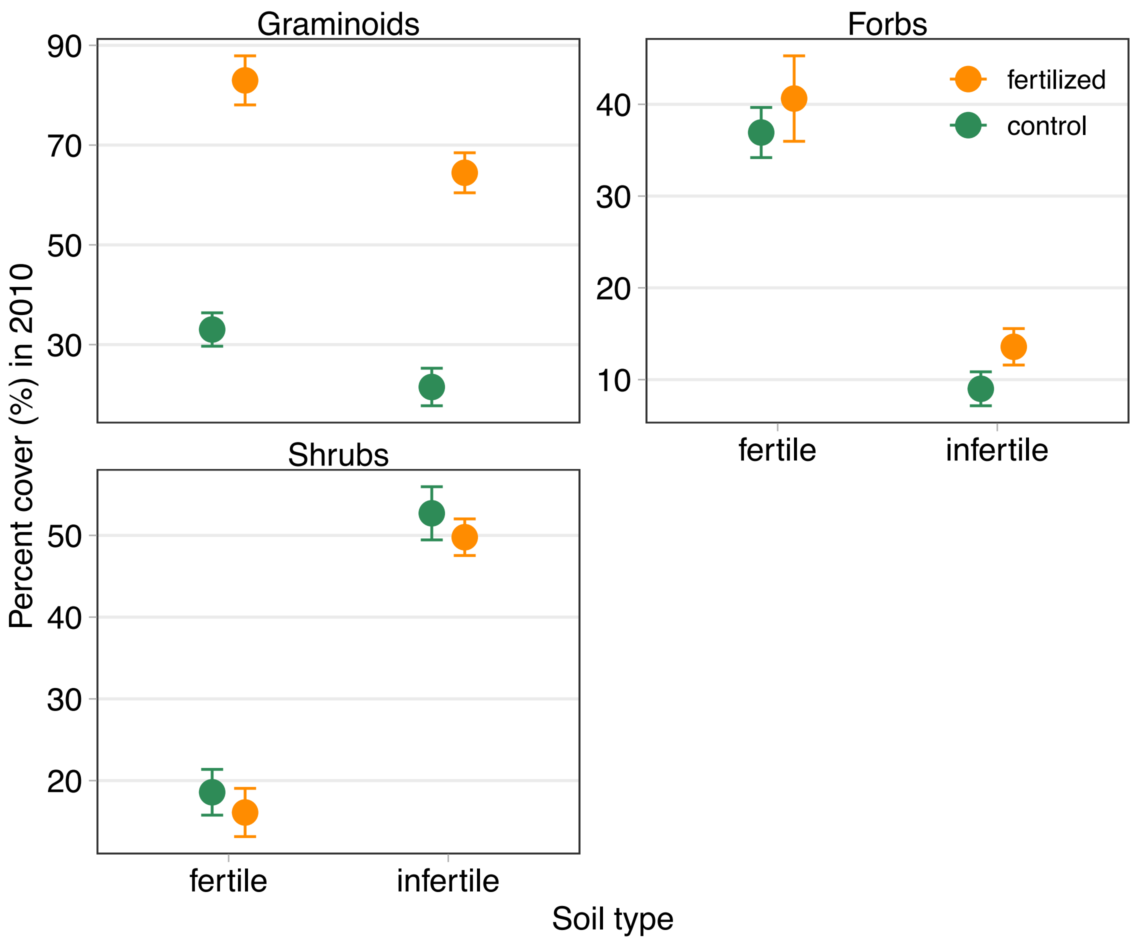

Figure S4: Total cover of bryophytes and lichens in 2019 (values are displayed as means ± se).

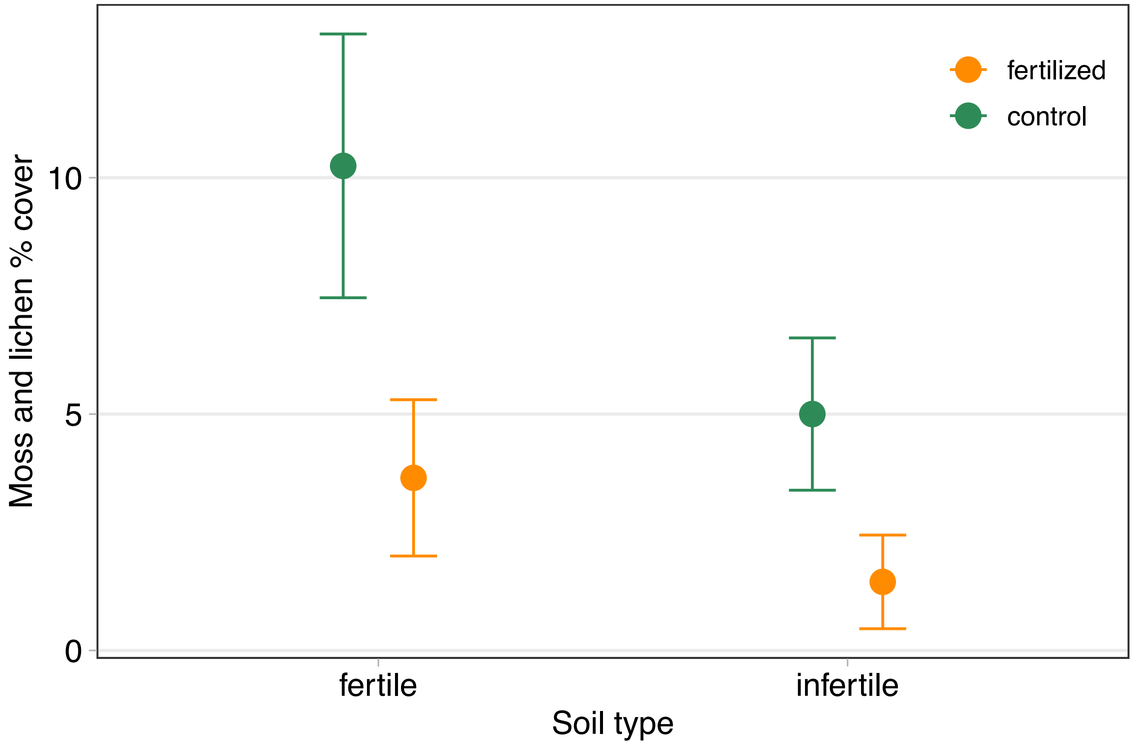

Figure S5: Relationship between species' traits and their probability to increase in cover between 2004 and 2019 by fertilization treatment, split by habitat type. (a) plant height (b) leaf carbon to nitrogen ratio (c) specific leaf area. Plots show fitted relationship with estimated standard errors.

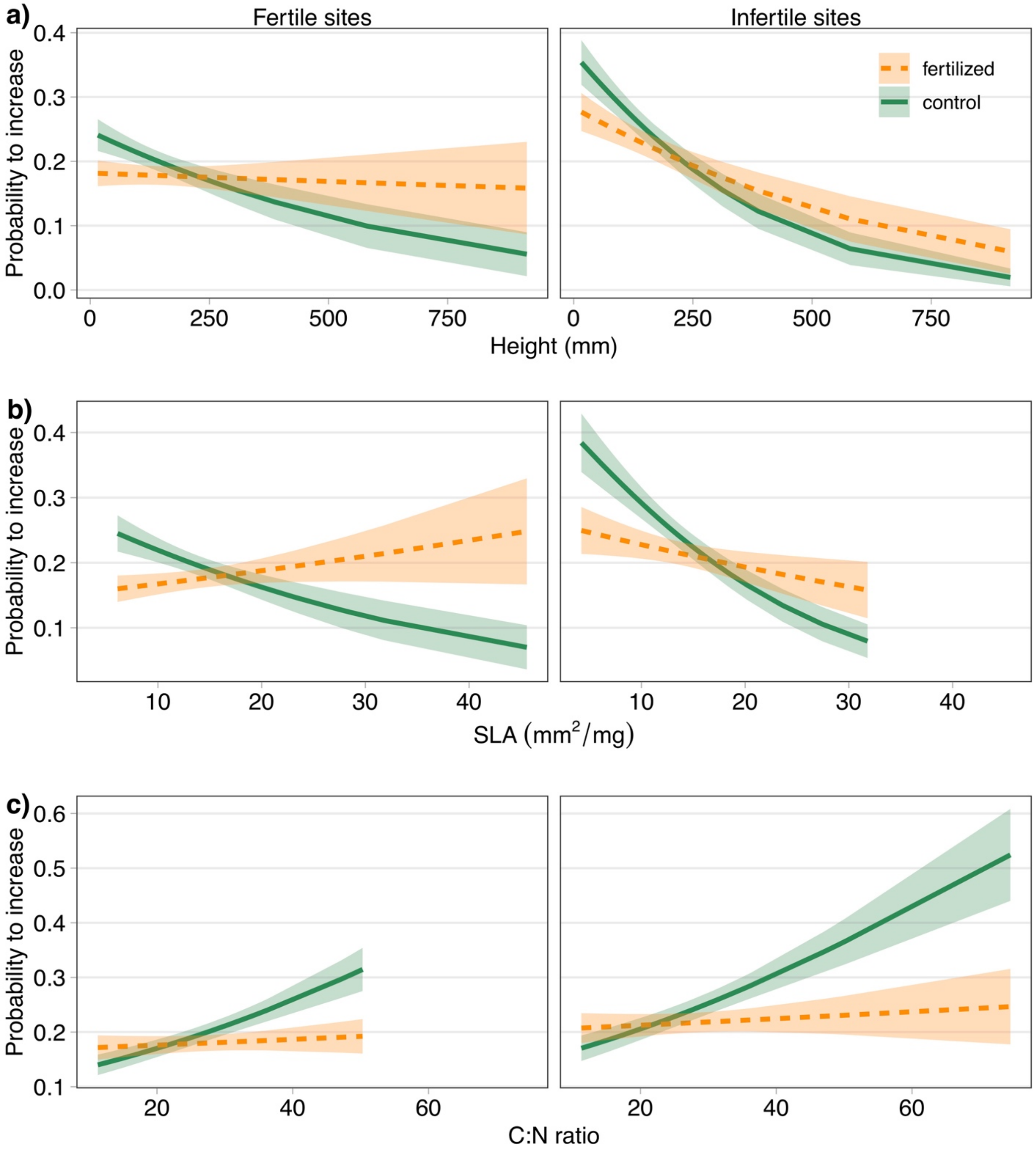

Figure S6: (a) Soil phosphorus concentrations and (b) soil C:N ratio in 2019 by fertilization treatment. Values are displayed as means ± se.

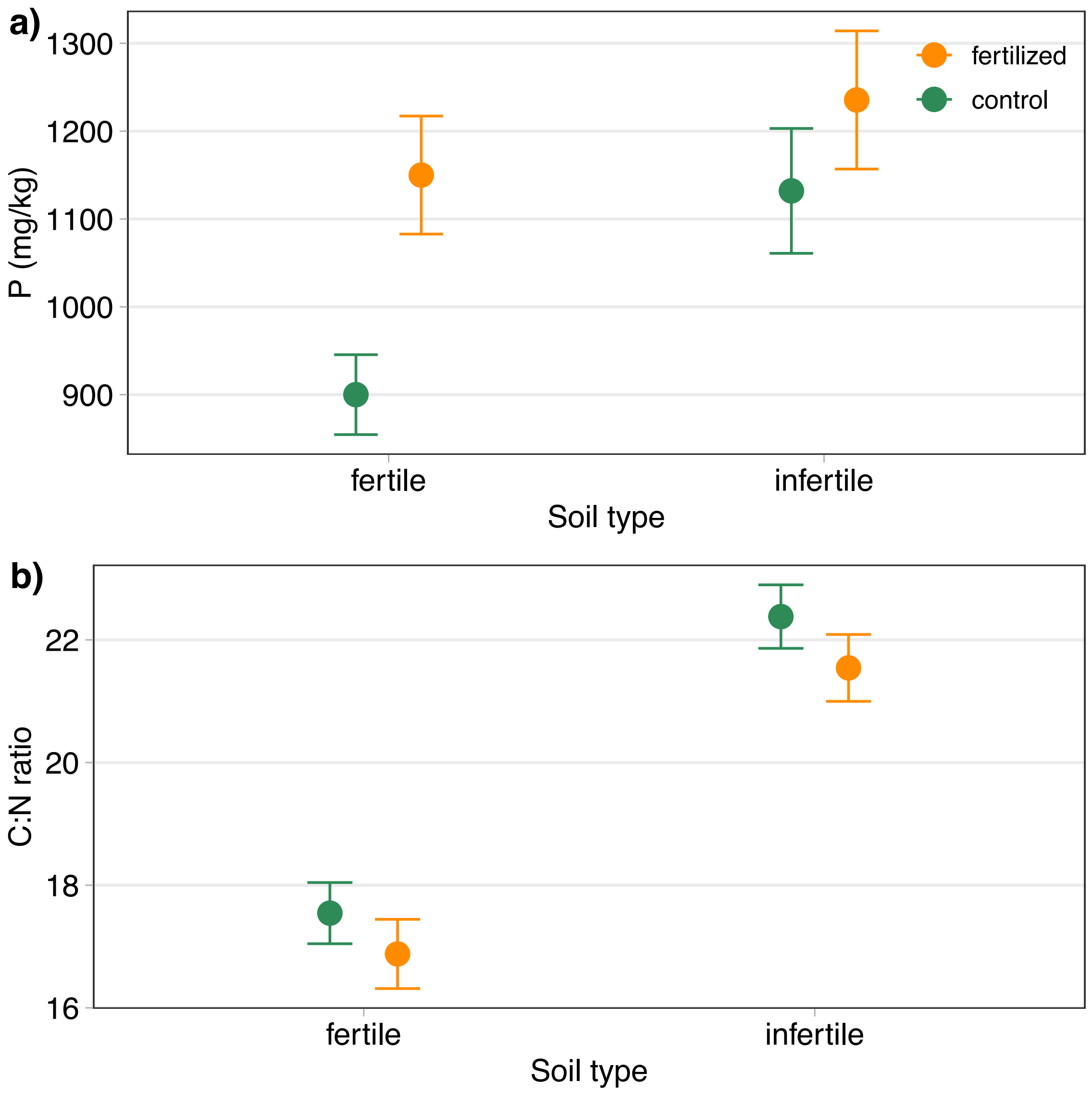

Supplemental Tables

Table S1: Comparisons of AICc values for selected models. Each "best fit" model is compared to a null model with only the intercept and random effects and a base no-interactions model that includes only main effects of fertilization, herbivory, and habitat (and trait for species trait models). ΔAICc values > 2 are bolded.

	selected model	AICc	df	null model ΔAICc	no-interaction ΔAICc
Height	trait + fertilization + herbivory + habitat + trait * fertilization + trait * habitat	1840	8	**25.0**	**3.23**
SLA	trait + fertilization + herbivory + habitat + trait * fertilization + trait * habitat	1895	8	**15.5**	**7.47**
C:N	trait + fertilization + herbivory + habitat + trait*fertilization	1852	7	**12.7**	**2.91**
Litter depth 2019	fertilization + herbivory + habitat + fertilization*herbivory	300.6	7	**52.4**	**3.56**
Soil P 2019	fertilization	1105	4	**12.2**	**3.59**
Soil C:N 2019	fertilization + habitat	341.7	5	**10.0**	**1.95**
Richness change (initial)	fertilization + habitat	358.6	5	**4.89**	**2.34**
Richness change (persistent)	herbivory + habitat	352.8	5	1.72	**2.31**
Turnover (initial)	fertilization + herbivory + habitat + fertilization *herbivory	-162.5	7	**30.2**	**2.40**
Turnover (persistent)	fertilization + habitat	-64.4	5	**16.6**	1.55
Gram cover change (persistent)	herbivory + habitat + herbivory*habitat	639.9	6	**4.70**	**2.40**
Forb cover change (persistent)	fertilization + herbivory + habitat + fertilization *habitat + herbivory*habitat	727.5	8	**38.8**	**15.6**
Shrub cover change (persistent)	herbivory	742.2	4	**5.9**	**4.08**
Moss + Lichen cover 2019	fertilization + habitat	574.8	5	**6.3**	1.0
Gram cover 2010 only	fertilization + habitat	678.1	5	**90.37**	0.7
Forb cover 2010 only	fertilization + habitat	642.2	5	**12.6**	1.6
Shrub cover 2010 only	herbivory + habitat	6.29.2	5	**26.0**	1.08

Table S2: As a robustness check to complement our generalized linear mixed model models of trait-based probability to increase, we modeled cover change from 2004 to 2019 of the species present in the plots with a linear mixed model. We used a standard model for all traits, with fixed effects of habitat, fertilization treatment, herbivory, and trait, and the interactions between 1. fertilization treatment and trait and 2. fertilization treatment and habitat. Effects were consistent between these models and the probability to increase models.

	Intercept	Trait	Fertilized	Herbivory	Habitat	Trait * Fertilized	Trait * Habitat
Height	-0.85 ± 0.44	0.16 ± 0.38	-1.16 ± 0.46	0.06 ± 0.46	1.4 ± 0.47	0.56 ± 0.42	-0.42 ± 0.43
SLA	-0.84 ± 0.43	0.25 ± 0.41	-0.98 ± 0.45	0.07 ± 0.45	1.3 ± 0.46	0.60 ± 0.50	-0.34 ± 0.52
C:N	-0.91 ± 0.45	-0.15 ± 0.45	-1.12 ± 0.46	0.06 ± 0.46	1.48 ± 0.47	-0.63 ± 0.51	0.19 ± 0.52

Table S3: Change in cover by species, values presented as cover (number of plots present).

	Fertile sites				Infertile sites			
	control		fertilized		control		fertilized	
Species	2004	2019	2004	2019	2004	2019	2004	2019
Equisetum pratense	0.1 (2)	0.2 (2)	0.1 (2)	0.2 (2)	0.1 (1)	0.9 (2)	0.3 (2)	2.9 (3)
Equisetum scirpoides	0.6 (9)	0.2 (4)	1.2 (11)	1 (6)	0.1 (2)	0.2 (2)	0.1 (2)	0.1 (2)
Equisetum variegatum	0.1 (2)	0 (0)	0.1 (2)	0 (0)	0 (0)	0 (0)	0 (0)	0 (0)
Equisetum variegatum	0.1 (2)	0 (0)	0.1 (2)	0 (0)	0 (0)	0 (0)	0 (0)	0 (0)
Astragalus alpinus	3 (12)	2.5 (10)	8 (14)	2 (11)	0.6 (5)	2 (6)	0.7 (8)	0.5 (2)
Astragalus frigidus	0.3 (1)	0.5 (1)	0.1 (1)	0.1 (1)	0 (0)	0.1 (1)	0 (0)	0 (0)
Bartsia alpina	0.1 (2)	0 (1)	0.4 (2)	0 (0)	0 (0)	0 (0)	0 (1)	0 (0)
Betula nana	5 (13)	3.5 (9)	3.7 (9)	1.4 (5)	9.8 (13)	13.5 (15)	10.4 (13)	11.1 (13)
Bistorta vivpara	3.3 (18)	3.2 (16)	5.9 (19)	2.9 (17)	0.4 (7)	0.9 (6)	0.4 (5)	1.2 (9)
Campanula rotundifolia	0 (0)	0 (0)	0 (0)	0.1 (1)	0 (0)	0 (0)	0 (0)	0 (0)
Cerastium alpinum alpinum	0.3 (3)	0 (0)	0.2 (2)	0 (1)	0 (0)	0 (0)	0.4 (2)	0.2 (1)
Cerastium alpinum alpinum	0.3 (3)	0 (0)	0.2 (2)	0 (1)	0 (0)	0 (0)	0.4 (2)	0.2 (1)
Draba daurica	0.1 (1)	0 (0)	0 (0)	0 (0)	0 (0)	0 (0)	0 (0)	0 (0)
Dryas octopetala	28.6 (17)	21.2 (16)	36 (19)	6.2 (12)	0 (0)	0 (0)	0 (0)	0 (0)
Erigeron uniflorum	0 (0)	0 (0)	0 (0)	0.1 (1)	0 (0)	0 (0)	0 (0)	0 (0)
Linnaea borealis	0 (0)	0 (0)	0 (0)	0 (0)	2 (5)	0 (0)	2.2 (5)	0.2 (1)
Lycopodium annotinum	0 (0)	0 (1)	0 (0)	0 (0)	0 (0)	0 (0)	0.8 (1)	0 (0)
Minuartia biflora	0.1 (2)	0 (0)	0 (1)	0 (0)	0 (0)	0 (0)	0 (0)	0 (0)
Oxyria digyna	0.4 (3)	0.1 (2)	0.5 (1)	0.2 (2)	0 (0)	0 (0)	0 (0)	0 (0)
Parnassia palustris	0.1 (1)	0.1 (1)	0 (0)	0 (0)	0 (0)	0 (0)	0 (0)	0 (0)
Pedicularis lapponica	0.8 (10)	0.3 (3)	0.1 (2)	0.1 (2)	0.3 (5)	0.2 (1)	0.2 (2)	0.2 (2)
Pseudorchis albida	0 (1)	0.1 (1)	0.1 (1)	0 (0)	0 (0)	0 (0)	0 (0)	0 (0)
Pseudorchis albida	0 (1)	0.1 (1)	0.1 (1)	0 (0)	0 (0)	0 (0)	0 (0)	0 (0)
Pyrola minor	0 (1)	0 (0)	0 (0)	0 (0)	0 (0)	0 (0)	0 (0)	0 (0)
Ranunculus acris	0 (0)	0 (0)	0 (0)	0.1 (1)	0 (0)	0 (0)	0 (0)	0 (0)
Saussurea alpina	0.1 (2)	0.4 (2)	0.6 (4)	0.7 (5)	0 (1)	0 (0)	0 (1)	0.1 (1)
Saxifraga aizoides	0.1 (1)	0.8 (1)	0 (0)	0 (0)	0 (0)	0 (0)	0 (0)	0 (0)
Saxifraga oppositifolia	0.4 (5)	0.4 (1)	0.6 (3)	0 (0)	0 (0)	0 (0)	0 (0)	0 (0)
Silene acaulis	6.8 (6)	11.2 (6)	4.4 (5)	1.4 (4)	0 (0)	0 (0)	0 (0)	0 (0)
Solidago virguarea	0 (0)	1.4 (4)	0 (0)	1.6 (7)	0 (0)	0.5 (2)	0 (0)	2.5 (6)
Thalictrum alpinum	1 (9)	1.2 (7)	1.4 (9)	0.6 (4)	0 (0)	0 (0)	0.2 (4)	0.8 (3)
Tofieldia pusilla	0.2 (2)	1.8 (2)	0 (0)	1.4 (2)	0 (0)	1 (2)	0 (0)	2 (2)
Viola biflora	0.2 (4)	0 (0)	0.2 (3)	0.1 (1)	0 (0)	0 (0)	0 (0)	0 (0)
Anthoxanthum odoratum	0 (0)	0 (0)	0 (1)	0.4 (4)	0 (0)	0 (1)	0 (0)	0.2 (2)
Calamagrostis lapponica	0 (1)	0 (0)	0.1 (2)	0 (0)	0.9 (10)	0 (0)	1.1 (10)	0 (0)
Carex capillaris	2.1 (2)	0.2 (2)	0 (1)	0 (0)	0 (0)	0 (0)	0 (0)	0 (0)
Carex rupestris	6.9 (18)	3.1 (11)	3.6 (19)	0 (0)	0.2 (4)	0.1 (1)	0.1 (3)	0 (0)
Carex vaginata	6.9 (20)	4.2 (19)	13.4 (20)	5.3 (20)	3.9 (16)	5 (15)	3.6 (19)	4 (19)
Festuca ovina	3.8 (13)	3.5 (12)	5.4 (17)	12.4 (17)	1.6 (14)	3.7 (16)	5.3 (17)	4.6 (15)
Luzula arctica	0 (0)	0 (0)	0.1 (1)	0.1 (1)	0 (0)	0.2 (1)	0 (0)	0 (0)
Poa alpina	0 (1)	0.4 (4)	0.2 (2)	0.2 (1)	0 (0)	0.1 (1)	0 (0)	0 (0)
Andromeda polifolia	0.3 (4)	0.8 (3)	0.1 (1)	0 (0)	0 (0)	0 (0)	0 (0)	0 (0)
Arctostaphylos uva-ursi	0 (0)	0 (0)	0 (0)	0 (0)	0.1 (1)	0 (0)	1.1 (2)	0 (0)
Arctostaphylus alpina	0.3 (1)	0.2 (1)	0 (0)	0 (0)	4.2 (6)	1.2 (3)	0.9 (2)	0.8 (3)
Cassiope tetragona	4.1 (6)	2 (8)	5.3 (8)	2.6 (4)	1.4 (3)	1.2 (3)	1.7 (3)	0.3 (2)
Empetrum nigrum	13.7 (13)	9.8 (14)	15.4 (15)	9.1 (16)	59.4 (20)	63.6 (20)	68 (20)	62.2 (20)
Juniperus communis	0 (0)	0 (0)	0 (0)	0 (0)	0 (0)	0 (0)	0 (0)	0.8 (1)
Rhododentrum lapponicum	1.4 (3)	0.1 (1)	0.1 (2)	0.3 (2)	0 (0)	0 (0)	0 (0)	0 (0)
Salix hastata	1.1 (3)	0.7 (3)	1.3 (3)	0.6 (2)	1.6 (3)	1.5 (2)	4.3 (6)	2.2 (5)
Salix herbaceae	0 (0)	0 (0)	0 (0)	0 (0)	0.3 (3)	0 (0)	0.2 (2)	0 (0)
Salix lapponica	0.1 (1)	0 (0)	0.2 (1)	0.2 (1)	0 (1)	0 (0)	0 (0)	1.2 (1)
Salix myrtilloides	0.1 (1)	0.2 (1)	0 (1)	0 (0)	0 (0)	0 (0)	0 (0)	1 (2)
Salix reticulata	2 (10)	4.3 (11)	1.4 (7)	6.2 (8)	0 (0)	0 (0)	0 (0)	0 (0)
Vaccinium uliginosum	12.1 (15)	7 (13)	6.7 (12)	5 (11)	12.4 (17)	9.5 (14)	8.6 (16)	7.5 (15)
Vaccinium vitis-ideae	0.2 (4)	0.5 (3)	0.2 (4)	0.8 (3)	7.8 (19)	11 (18)	8.6 (20)	9.2 (15)
